# A Reinforced Perfluorosulfonic Acid Membrane with PE Mesh

**DOI:** 10.3390/membranes16050177

**Published:** 2026-05-17

**Authors:** Yiru Dou, Bihai Su, Ying Jin, Wen Zhang, Yue Wang, Yuxin Wang

**Affiliations:** 1 State Key Laboratory of Chemical Engineering and Low-Carbon Technology, Tianjin Key Laboratory of Membrane Science and Desalination Technology, School of Chemical Engineering and Technology, Tianjin University, Tianjin 300072, China; douyiru@tju.edu.cn (Y.D.);; 2 Hebei Gellec New Energy Science & Technology Co., Ltd., Handan 057150, China

**Keywords:** PE mesh, perfluorosulfonic acid, solution casting, composite membranes

## Abstract

Perfluorosulfonic acid (PFSA) membranes are a key component in many applications, but their low dimensional stability and mechanical strength can result in unsatisfactory device performance and a short life span. To effectively and economically mitigate these limitations with the lowest possible sacrifice of desirable properties, we report herein a PFSA membrane reinforced with a low-cost and easily available polyethylene (PE) mesh fabricated using a simple solution casting method. The high-strength and non-swellable mesh embedded in the PFSA matrix restricts its free swelling. As a result, the reinforced membrane shows a remarkably enhanced dimensional stability, lowering the areal swelling ratio to ~8% in water at 100 °C, in contrast to the ~58% of the unreinforced solution-cast membrane and ~44% of the melt-extruded commercial N117 membrane. Although the non-conductive PE mesh poses certain hindrances to proton transport, the reinforced membranes maintain ~94% of the proton conductivity of the pure PFSA membrane. Moreover, the mechanical strength of the reinforced membrane is enhanced to nearly three times that of the unreinforced one, reaching ~44 MPa. The incorporation of the PE mesh also leads to an enhanced resistance to oxidative corrosion and H_2_ gas crossover of the membrane. This research demonstrates a promising technological pathway for developing high-performance and cost-competitive PFSA membranes.

## 1. Introduction

Perfluorosulfonic acid (PFSA) is a key material in the area of energy storage and conversion that is critical for addressing the crucial problems of global warming and sustainable energy. Owing to its high ionic conductivity and excellent chemical stability, PFSA has found a wide variety of applications, especially in making proton exchange membranes (PEMs) for various electrochemical devices such as fuel cells [1,2,3,4], water electrolyzers [5,6,7], and flow batteries [8,9].

However, a PEM made of PFSA is not without drawbacks. One noticeable drawback of a PFSA membrane is that its high proton conductivity can only be realized under sufficient hydration [10,11]. But a highly hydrated PFSA membrane tends to swell excessively, which can result in several adverse effects. If the membrane swells a lot, the catalyst layer in the membrane electrode assembly (MEA) of an electrochemical cell may delaminate, resulting in an accelerated performance degradation [12,13]. The excessive swelling of PEMs may also generate uneven and alternating internal stresses, which can prompt crack formation and thus reduce the mechanical stability of the membrane [14,15]. Moreover, a highly swellable membrane would enable a rapid crossover of fed or produced molecules, resulting in reduced fuel efficiency in direct methanol fuel cells [16,17,18] or decreased gas purity in PEM water electrolyzers [19,20,21].

Different strategies have been proposed to mitigate excessive swelling and augment the dimensional stability of PFSA membranes. The mixed matrix membrane (MMM) strategy was widely attempted by dispersing a different variety of fillers into the PFSA matrix [22,23,24], which, however, often cannot suppress membrane swelling sufficiently. Chemical crosslinking [25,26,27] and polymer blending [28,29] were also attempted, but this can result in either unsatisfactory swelling suppression or an unacceptable decrease in proton conduction. In addition, the “pore-filling” approach was employed to restrict the swelling of PFSA and enhance the mechanical strength of the resulting membrane by filling PFSA resin into the pores of a microporous membrane that was mechanically strong and non-swelling [30,31,32]. The commercial Gore-Select membrane made by impregnating expanded PTFE (ePTFE) with PFSA proved the efficacy of the “pore-filling” strategy. But an ePTFE film suitable for impregnation is not easily available, and a complete impregnation of the PFSA polymer into the microporous membrane can be technologically complex. Therefore, the search is still ongoing for more satisfactory strategies to enhance the mechanical strength and dimensional stability of PFSA-based membranes.

Following our previous study on reinforcing PFSA membranes with a fiberglass cloth [33], herein we report a new method of using polyethylene (PE) mesh to strengthen a PFSA membrane and limit its excessive swelling. PE is among the cheapest synthetic polymers and is easily processable, yet it is highly resistant to chemical corrosions and can exhibit very high mechanical strength. In addition, PE mesh has large and straight-through pores that can be easily filled with PFSA resin. These properties make PE mesh a potentially good alternative to reinforce a PFSA membrane. The PE-reinforced PFSA membranes were fabricated using a simple solution casting method. The swelling properties, mechanical strength, proton conductivity, H_2_ gas crossover and chemical stability of the reinforced membranes were tested and compared with those of an unreinforced solution-cast membrane and/or a melt-extruded commercial N117 membrane.

## 2. Experimental Section

### 2.1. Materials and Chemicals

Perfluorosulfonic acid resin powder (PFSA, 1.03 mmol/g, 1.98 g/cm^3^) was purchased from Shandong Dongyue Future Hydrogen Energy Material Co., Ltd. (Zibo, China). Polyethylene mesh (PE mesh, 100 mesh and 120 mesh, with pore sizes of 150 μm and 125 μm, respectively) was obtained from Anping Xingtai Mesh Co., Ltd. (Hengshui, China). Deionized water (H_2_O, >1 MΩ·cm) was obtained from Tianjin Yongqingyuan Distilled Water Shop (Tianjin, China). Hydrogen peroxide (H_2_O_2_, AR, 30 wt.%) was obtained from Tianjin Jiangtian Chemical Technology Co., Ltd. (Tianjin, China). Sulfuric acid (H_2_SO_4_, AR, 98%) was obtained from Rionlon (Tianjin) Pharmaceutical Chemistry Co., Ltd. (Tianjin, China). Ferrous sulfate heptahydrate (FeSO_4_·7H_2_O, AR) was purchased from Tianjin Kermel Chemical Reagents Co., Ltd. (Tianjin, China). Hydrogen (H_2_, 99.999%) and nitrogen (N_2_, 99.999%) gases were purchased from Tianjin Boliming Technology Co., Ltd. (Tianjin, China). Pt/C catalyst (0.5 mg/cm^2^, HCP120) was purchased from Shanghai Hesen Electric Co., Ltd. (Shanghai, China). Commercial Nafion 117 membrane was purchased from Chemours (Shanghai) Co., Ltd. (Shanghai, China).

### 2.2. Membrane Preparation

A 5 wt.% PFSA solution was prepared by dissolving 1 g of PFSA powder into 19 g DMF under reflux at 120 °C for 2 h. Two PE meshes, with 100 and 120 mesh counts respectively, were washed successively with absolute ethanol and deionized water before drying for future use. To prepare a reinforced membrane, a portion of the casting solution was first drop-coated onto a glass plate, followed by placing a treated PE mesh on top of the solution layer. An additional casting solution was then added dropwise onto the PE mesh and allowed to stand for 12 h to ensure full solution impregnation. The composite was subsequently dried in a vacuum oven at 80 °C for 12 h. After cooling to room temperature, the resultant reinforced membranes were immersed in deionized water, carefully peeled off from the glass plate with tweezers, and stored in deionized water for later use. The schematic diagram of the preparation process is shown in Figure S4. The PFSA membranes reinforced with 100-mesh and 120-mesh PE are signified as 100PE-PFSA and 120PE-PFSA, respectively. For comparison purposes, a pure PFSA membrane without PE mesh was also cast under identical conditions and is signified as PFSA.

### 2.3. Characterizations and Property Tests

The morphology and microstructure of the membranes were imaged using a scanning electron microscope (SEM, 8100, Hitachi, Tokyo, Japan) with an accelerating voltage of 3 kV. Prior to characterization, all samples were thoroughly dried at 80 °C for 24 h. To observe the cross-section, the membrane was cryo-fractured in liquid nitrogen to ensure a clean fracture surface. All samples were fixed onto the stage using a conductive adhesive and coated with a thin layer of Pt via ion sputtering to enhance conductivity. Infrared spectra of the membranes were recorded using an attenuated total reflection Fourier transform infrared (ATR-FTIR) spectrophotometer (FTIR-650, Tianjin Gangdong Sci. & Tech. Co., Ltd., Tianjin, China). The dried samples were placed on a ZnSe crystal stage, and spectra were collected over a range of 4000–650 cm^−1^ with a resolution of 4 cm^−1^ and 32 scans. A background scan was performed before each measurement to subtract the influence of ambient H_2_O and CO_2_.

The water uptake ($W_{U}$) and swelling ratios in terms of area (A_SR_) and thickness (T_SR_) were determined by measuring the changes in membrane mass and dimensions between the dry and fully hydrated states. Rectangular specimens (10 mm × 30 mm) were dried to a constant weight in a vacuum oven at 80 °C for 12 h to obtain their initial mass (W_dry_), area (A_dry_), and thickness (T_dry_). Subsequently, the samples were immersed in deionized water at various temperatures for 1 h. Upon removal, surface water was quickly removed with filter paper, and the mass (W_wet_), area (A_wet_), and thickness (T_wet_) of the swollen membranes were immediately measured. For each condition, three parallel samples were tested to obtain an average value. These parameters were calculated as follows:

(1) $$WU\left(\%\right)=\frac{W_{wet}-W_{dry}}{W_{dry}}\times 100\%$$

(2) $$T_{SR}\left(\%\right)=\frac{T_{wet}-T_{dry}}{T_{dry}}\times 100\%$$

(3) $$A_{SR}\left(\%\right)=\frac{A_{wet}-A_{dry}}{A_{dry}}\times 100\%$$

Mechanical properties were evaluated at ambient temperature using a ZCW-5000 testing system (Jinan Zhongchuang Industrial Test System Co., Ltd., Jinan, China). Dumbbell-shaped specimens, featuring a 4 mm wide central portion and a length of 30 mm, were prepared using a cutting die. During the test, the specimen was fixed between the fixtures and stretched at a constant rate of 5 mm·min^−1^. The corresponding stress–strain curves were obtained to analyze the mechanical performance of the membranes.

The proton conductivity of the membranes was measured using electrochemical impedance spectroscopy (EIS). Before measurements, the membranes were pretreated by being successively immersed in 5 wt.% H_2_O_2_, deionized water, 1 M H_2_SO_4_, and deionized water at 80 °C for 1 h each. Rectangular specimens were then mounted in a four-electrode fixture connected to an MTS-740 membrane test system and a PARSTAT 2273 electrochemical workstation. The impedance spectra were recorded from 1 Hz to 2 MHz with an oscillating voltage of 10 mV. The proton conductivity (σ, S/cm) was calculated according to the following equation:

(4) $$\sigma =\frac{L}{RA}$$

where L and A are the thickness and effective area of the membrane, respectively.

The hydrogen permeability of the membranes was determined via an in situ electrochemical method as described in reference [34]. During the measurement, the membrane was clamped between two electrodes and fully immersed in the solution at room temperature, as shown in Figure S3. A constant current density of 200 mA·cm^−2^ was applied to the hydrogen evolution reaction electrode using a DC power supply to generate H_2_. The evolved H_2_ diffused through the membrane to the hydrogen oxidation reaction electrode on the opposite side. At a sufficiently high positive potential, the H_2_ was completely oxidized at the detection electrode, where the limiting current density (J_l_, mA·cm^−2^) was determined by the rate of hydrogen permeation. Linear sweep voltammetry (LSV) was performed using an electrochemical workstation with a scan range of 0–1.5 V at a scan rate of 5 mV·s^−1^. The J_l_ was obtained by fitting the polarization curves and taking the intercept of the plateau region. The H_2_ permeation flux (J_H_, L cm^−2^ s^−1^) and the hydrogen permeation rate (Φ_H_, L cm cm^−2^ s^−1^) were calculated using the following equations:

(5) $$J_{H}=\frac{J_{l}\times V_{m}}{2F}$$

(6) $$\Phi_{H}=J_{H}\times \delta_{m}$$

where F is the Faraday constant (96,485 A·s·mol^−1^), V_m_ is the molar volume of gas at STP (22.4 L mol^−1^), and δ_m_ is the membrane thickness (cm).

The chemical oxidative stability of the membranes was tested in Fenton’s reagent (2 ppm FeSO_4_ and 3% H_2_O_2_) at 80 °C. Membrane samples (1 × 1 cm) were dried and weighed before being immersed in the solution for specified time intervals. After the oxidative treatment, the samples were washed repeatedly with deionized water and dried to a constant weight. The oxidative stability was signified by the remaining mass fraction, calculated as the average of three parallel measurements.

## 3. Results and Discussion

**Figure 1:**
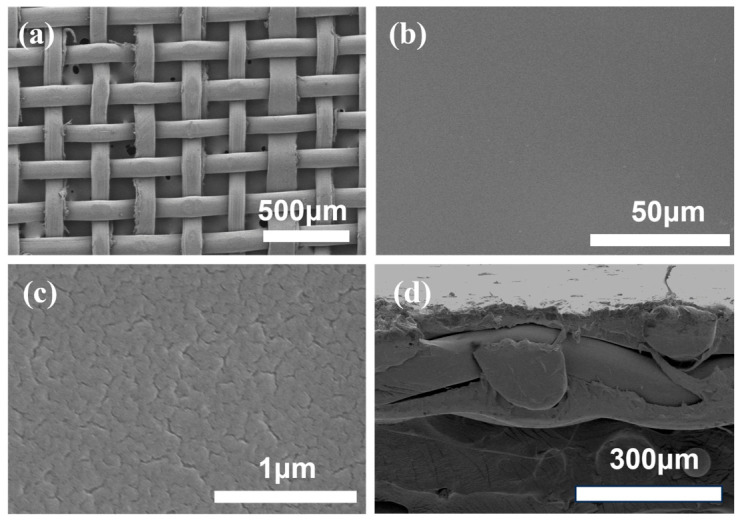
SEM images of the PE mesh and its reinforced PFSA membrane: (**a**) the top view of the 120^#^ PE mesh and (**b**,**c**) the surface views and (**d**) cross-sectional view of the reinforced PFSA membrane.

The typical morphology of the PE meshes used and the PE-reinforced PFSA membranes is illustrated in Figure 1. The PE mesh is plain woven with structurally well-defined straight-through pores (Figure 1a), which can facilitate the complete impregnation of the PFSA resin and proton transport in the resultant membrane. The simple solution casting membrane fabrication led to a reinforced PFSA membrane with a flat and dense surface (Figure 1b,c). The tiny fractures on the membrane surface (Figure 1c) may result from the internal stress generated during the dehydration of a heterogeneous PFSA top layer [35]. Upon drying, the hydrophilic phase of sulfonate groups in PFSA would lose water and shrink, while the neighboring hydrophobic backbone would not, thus generating a stress. The cross-sectional view of the reinforced PFSA membrane (Figure 1d) indicates that the PE mesh was totally wrapped in the PFSA resin and properly set in the middle of the membrane, enabling its best mechanical support to the PFSA membrane. This observation is consistent with the SEM images of the 100-mesh PE shown in Figure S1.

**Figure 2:**
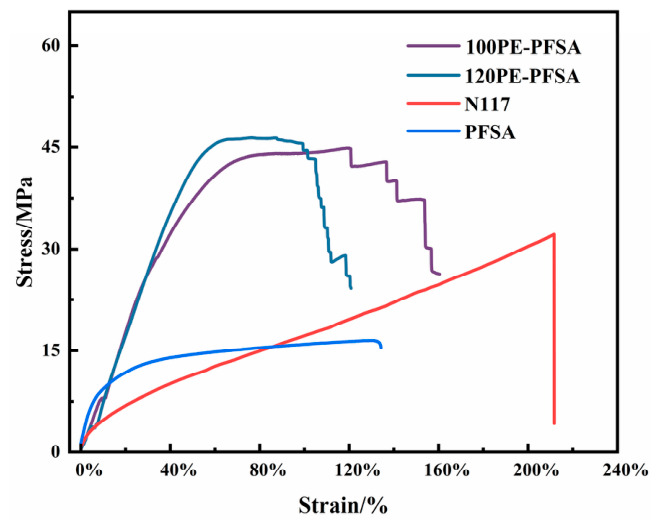
Stress–strain curves of PE-reinforced PFSA membranes.

An immediate consequence of the reinforcement with the PE mesh is the significantly enhanced breaking strength of the resultant membrane, as revealed in Figure 2. The PFSA membranes reinforced with 100^#^ and 120^#^ PE mesh showed an average breaking strength of 44 MPa, which was 2.8 times that of the unreinforced solution-cast PFSA membrane and 1.33 times that of the melt-extruded commercial N117 membrane. The PE-reinforced PFSA membranes maintained a breaking strain higher than 100%, retaining the good toughness and flexibility of the unreinforced counterparts. It is noticed that the PFSA membranes reinforced with the PE mesh did not break suddenly, like the case of the unreinforced membranes, as reflected by the step-like part of the stress–strain curves of the corresponding membranes. This can probably be attributed to the successive, rather than simultaneous, breaking of latitudinal fibers in the PE mesh. The inconsistency in the step-like character of the two membranes may indicate their differences in adhesion between the PFSA matrix and PE mesh or impregnation quality.

**Figure 3:**
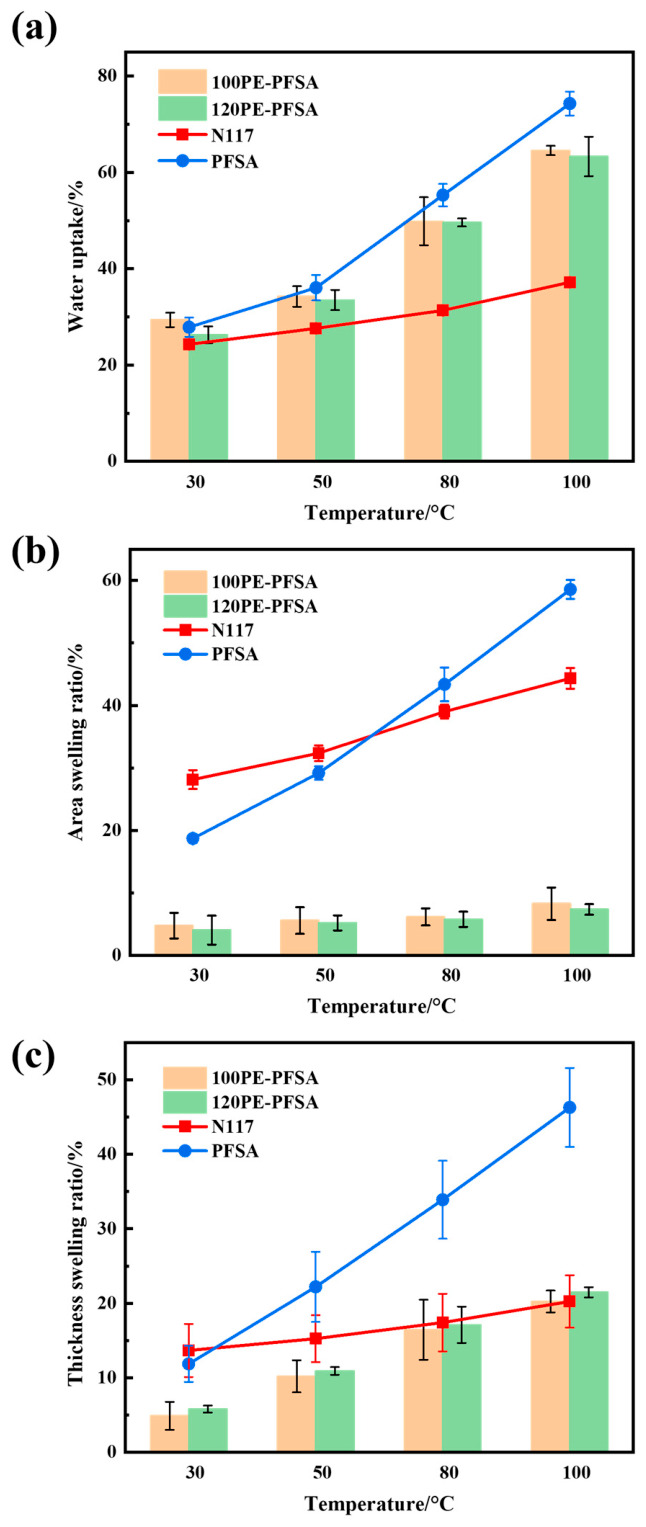
Water absorption and dimensional changes in PE-reinforced PFSA membranes: (**a**) water uptake, (**b**) area swelling, and (**c**) thickness swelling.

Figure 3 shows the water absorption and dimensional changes in reinforced PFSA membranes at different temperatures and compares these properties with those of the unreinforced solution-cast membrane and melt-extruded N117 membrane. As expected, the water uptakes of all membranes increased with temperature (Figure 3a). The PE-reinforced membranes had slightly lower water uptake when compared with the unreinforced solution-cast membrane. This is easily understandable since the embedded PE meshes can hardly absorb water, so that the relative weight change in PE-reinforced membranes was smaller after water soaking. However, the melt-extruded N117 membrane absorbed much less water than the solution-cast membranes, especially at higher temperatures. The lower water uptake of the N117 membrane can be explained by a higher degree of crystallinity that arises from its fabrication by melting extrusion [36,37].

The PFSA membranes reinforced with the PE mesh exhibited not only a considerably restricted area swelling ratio but also a very small change in the area swelling ratio with temperature. In contrast to the unreinforced membrane, which exhibited a change in the area swelling ratio from 19% at 30°C to 58% at 100°C, the two reinforced membranes exhibited area swelling ratios varying from about 5% to 8% in the same temperature range (Figure 3b), revealing the very effective restriction by the PE mesh of the areal dimensional change in the PFSA membrane. This can be attributed to a mechanical force exerted by the high-strength and non-swellable mesh on the PFSA matrix that restricts its free swelling in the lateral direction [38,39,40]. The chemical interactions between the PFSA matrix and PE mesh should not be significant, considering the rather chemically inert and low surface energy nature of PE. The embedded PE mesh also posed noticeable limitations on the swelling of reinforced membranes in the thickness direction, though it was not as strong as the limitation in the areal direction (Figure 3c). Consequently, the thickness swelling ratios of reinforced membranes were noticeably higher than the membranes’ corresponding area swelling ratios (Figure 3b). These greatly restricted swellings and enhanced dimensional stability of the reinforced PFSA membranes are very desirable for their use in various electrochemical cells to ensure their proper assembly and stable operation.

**Figure 4:**
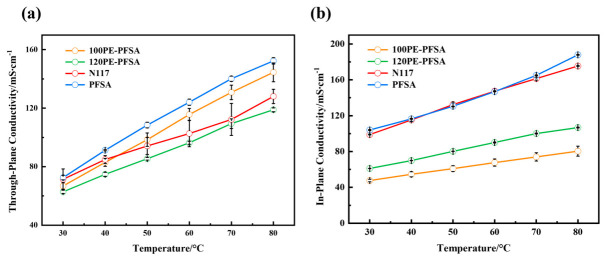
The proton conductivity vs. temperature of PFSA membranes: (**a**) through-plane conductivity and (**b**) in-plane conductivity.

Figure 4 illustrates the proton conductivity vs. temperature of different PFSA membranes. The PE-reinforced membranes showed lower through-plane conductivity compared with the unreinforced solution-cast PFSA membrane (Figure 4a). This is expected since the PE meshes are a proton insulator, and their embedment in the PFSA matrices would hinder the total proton conduction to a certain extent. For the same reason, membrane 120PE-PFSA had lower through-plane conductivity compared to membrane 100PE-PFSA, since the mesh embedded in the former had a smaller area of openings and thus a higher blockage of proton conduction. It is noted that the through-plane conductivities of the two mesh-reinforced membranes were 144 mS/cm and 119 mS/cm at 80°C, reaching respectively 94% and 78% of their unreinforced counterpart. This well-retained through-plain conductivity is important for a high-performance electrochemical device, while the small conductivity losses should be deemed as quite acceptable sacrifices, especially considering the remarkable increase in the enhancement of the dimensional stability of the PFSA membranes.

The proton conduction in the in-plane direction of PE-reinforced membranes dropped considerably in comparison with that of the pure PFSA membranes (Figure 4b). The in-plane conductivity of membrane 100PE-PFSA was 80 mS/cm at 80 °C, only 43% that of the solution-cast pure PFSA membrane at the same temperature. The large decreases in conductivity in the in-plane direction, i.e., the areal direction, were due to a strong blockage of the proton transport by the embedded reinforcing PE meshes. Fortunately, such a noticeable drop in in-plane conductivity is not as serious a problem as a drop in through-plane conductivity, because it is the through-plane conductivity of the membrane that is more relevant to the performance of most electrochemical devices.

The H_2_ barrier properties of the three solution-cast PFSA membranes (Figure S2) are presented in Table 1. In terms of the H_2_ crossover limiting the current or permeation flux, the PE-reinforced membranes were obviously better gas barriers over the pure PFSA membrane. Because the two reinforced membranes were much thicker, a comparison of the H_2_ gas permeability with the unreinforced membrane would also be relevant and meaningful for evaluating the effects of the embedded PE mesh on the H_2_ crossover of the resultant membranes. It turns out that the H_2_ permeability Φ_H_ of the two reinforced membranes was also lower, which is probably due to the higher gas resistance of PE over PFSA and the suppressed swelling of PFSA by the PE mesh. This speculation is supported by the permeability Φ_H_ data of the two reinforced membranes. Since the 120^#^ PE mesh was more densely woven than the 100^#^ PE mesh, the former could better resist H_2_ transport through itself and better restrict PFSA swelling, thus leading to the lower gas permeability of the 120PE-PFSA membrane. 

**Figure 5:**
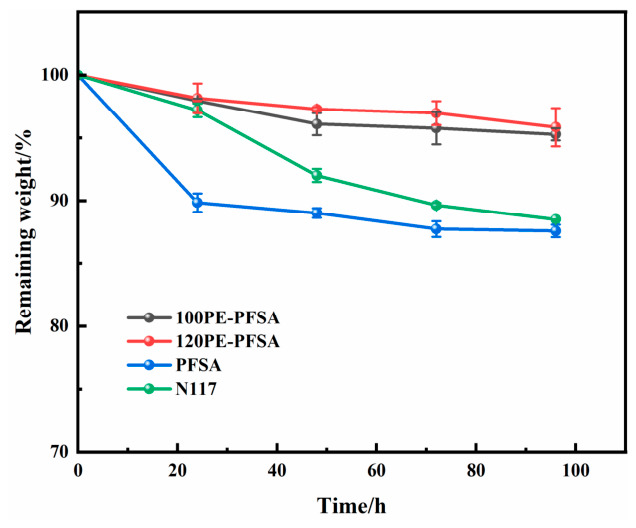
The weight change in different PFSA membranes during the Fenton test.

It is interesting that the PE-reinforced PFSA membranes appeared to better withstand Fenton oxidation compared to the unreinforced membranes. It is seen from Figure 5 that the mass losses were only 4.15% and 4.72% respectively for the two PE-reinforced membranes after 96 h of Fenton oxidation, in contrast to the 11.5% and 12.4% mass losses of the two pure PFSA membranes. Presumably, PE is better than PFSA at resisting the attack by the hydroxyl free radicals generated from Fenton reactions. Accordingly, the PE mesh inside the PFSA membrane not only degraded at a lower speed itself but also mitigated the degradation of PFSA by dividing the PFSA matrix into smaller regions and physically hindering the migration of free radicals and the propagation of chain degradation reactions. It is also possible that the increased chemical stability arises from the hindered diffusion of Fenton’s reagent into the reinforced membranes because of their considerably lower swelling ratio.

## 4. Conclusions

A PFSA membrane reinforced with an inexpensive and easily available PE mesh is fabricated using a simple solution casting method. The PE reinforcement enables a significantly enhanced dimensional stability of the resultant membrane by suppressing the membrane areal swelling ratio to less than 9% in water at 100 °C, while up to 94% of the through-plain proton conductivity of the pure PFSA membrane at 80 °C can be retained. The PE reinforcement can also lead to remarkably increased mechanical strength, lower H_2_ gas crossover permeability, and noticeably enhanced chemical stability. This research presents a promising alternative for developing high-performance and cost-competitive ionic conductive membranes for energy storage and conversion.

## Figures and Tables

**Table 1:** A summary of the H_2_ gas crossover limiting current density J_L_, permeation flux J_H_ and permeability Φ_H_ of different membranes at room temperature.

Membrane	Thickness */µm	J_l_/mA cm^−2^	J_H_ × 10^8^/L cm^−2^ s^−1^	Φ_H_ × 10^10^/L cm cm^−2^ s^−1^
PFSA	160	3.07	35.6	57.0
100PE-PFSA	320	1.35	15.7	50.1
120PE-PFSA	300	0.977	11.3	34.0

* Membrane thickness at room temperature.

## Data Availability

The original contributions presented in this study are included in the article. Further inquiries can be directed to the corresponding authors.

## Supplementary Materials

The following supporting information can be downloaded at: https://www.mdpi.com/article/10.3390/membranes16050177/s1, Figure S1: SEM images of PE mesh and its reinforced PFSA membrane: (a) the top view of 100# PE mesh; (b,c) the surface views and (d) cross-sectional view of the reinforced PFSA membrane; Figure S2: Gas barrier property tests of different membranes. (a,c,e) LSV curves for hydrogen crossover measurement, and (b,d,f) the corresponding magnified views of the regions in the left panels. (a,b) 100PE-PFSA, (c,d) 120PE-PFSA, (e,f) PFSA; Figure S3: Schematic diagram of the in-situ method for H2 permeability of the membrane(M1); Figure S4: Schematic diagram of PE mesh/PFSA composite membrane preparation process. Reference [34] is cited in Supplementary Materials.
