# Supplementary material for: A Reinforced Perfluorosulfonic Acid Membrane with PE Mesh
Source: Membranes (Basel). 2026 May 17;16(5):177. doi: 10.3390/membranes16050177 (PMC13208155; doi:10.3390/membranes16050177)
Supplement: Supplementary file 1 [file membranes-16-00177-s001.zip › membranes-4292655-supplementary.pdf]

Supporting Information

A Reinforced Perfluorosulfonic Acid Membrane with PE Mesh

Yiru Dou[1], Bihai Su[1,2], Ying Jin[1], Wen Zhang[1], Yue Wang[1*], Yuxin Wang[1*]

1 State Key Laboratory of Chemical Engineering and Low-Carbon Technology, Tianjin Key Laboratory of Membrane Science and Desalination Technology, School of Chemical Engineering and Technology, Tianjin University, Tianjin 300072, China

2 Hebei Gellec New Energy Science & Technology Co., Ltd., Handan 057150, China

*Corresponding authors

tdwy75@tju.edu.cn (Y.W.); yxwang@tju.edu.cn (Y.W.)

Results and discussion

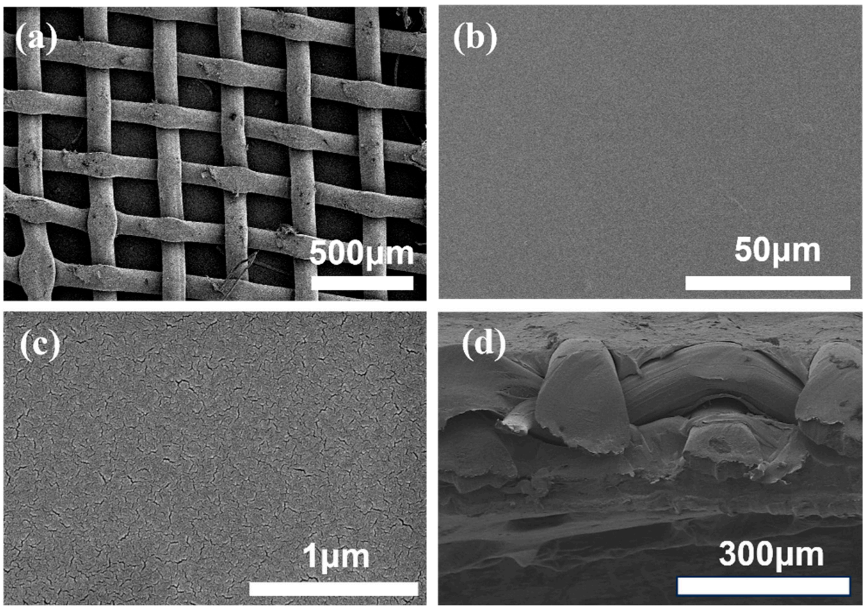

Figure S1. SEM images of PE mesh and its reinforced PFSA membrane: (a) the top view of 100# PE mesh; (b,c) the surface views and (d) cross-sectional view of the reinforced PFSA membrane.

Figure S1 presents the surface and cross-sectional morphologies of the 100-mesh PE mesh and its corresponding reinforced PFSA membrane. The SEM in Figure S1a illustrates the characteristic woven structure and well-defined pore distribution of the PE mesh. Through the solution casting method, a flat and dense reinforced membrane was successfully fabricated (Figure S1b,c). Notably, the cross-sectional view in Figure S1d demonstrates that the PE mesh is thoroughly encapsulated within the PFSA matrix, indicating excellent structural integration.

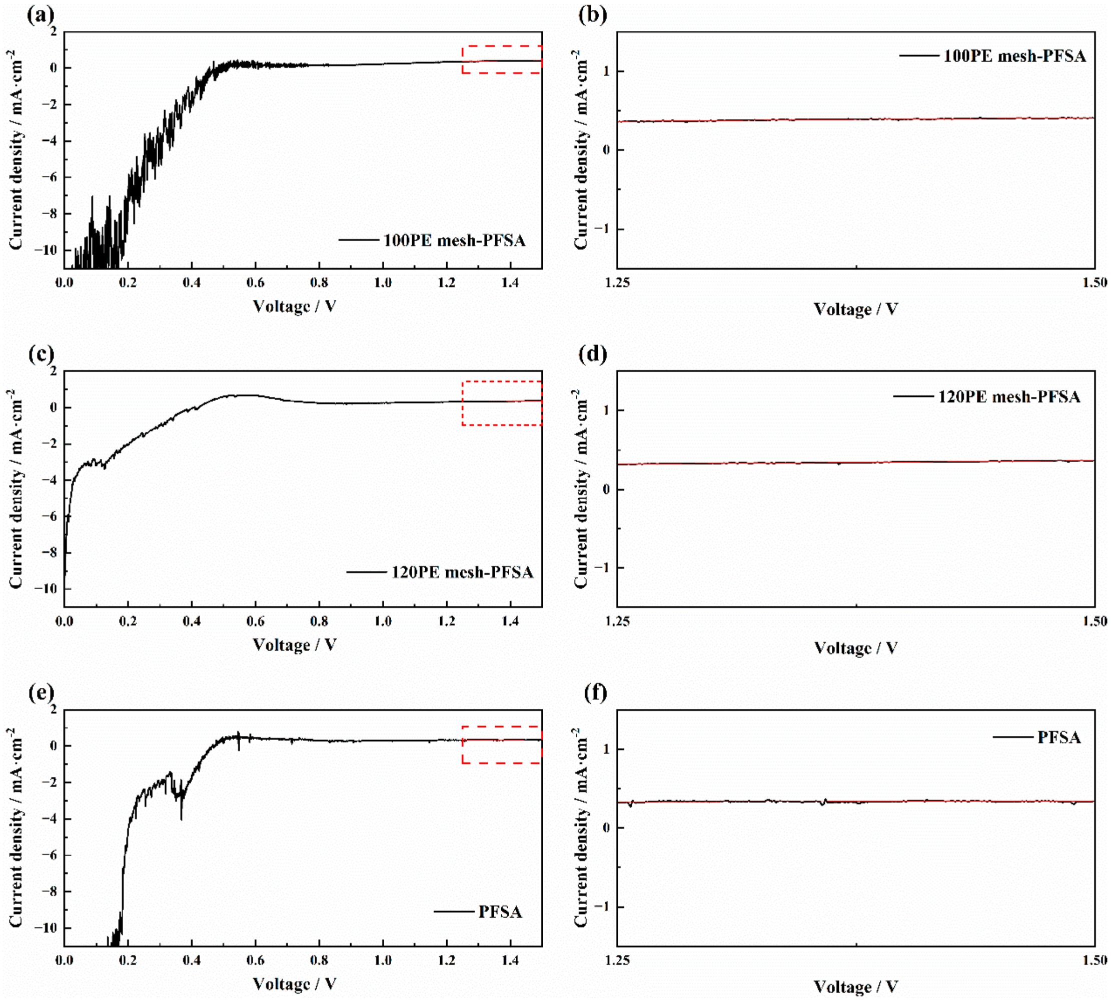

Figure S2. Gas barrier property tests of different membranes. (a, c, e) LSV curves for hydrogen crossover measurement, and (b, d, f) the corresponding magnified views of the regions in the left panels. (a, b) 100PE-PFSA, (c, d) 120PE-PFSA, (e, f) PFSA.

The gas barrier property tests of the membranes were conducted at room temperature using an in-situ electrochemical method, with results presented in Figure S2. The current density initially increases with the applied voltage before reaching a plateau. To determine the actual limiting current density, a linear fit was applied to the LSV curves within the potential range of 1.25–1.5 V. By extrapolating this fitted line to the vertical axis, the intercept was obtained. Subsequently, the hydrogen crossover rate and hydrogen permeability were calculated based on this derived limiting current density.

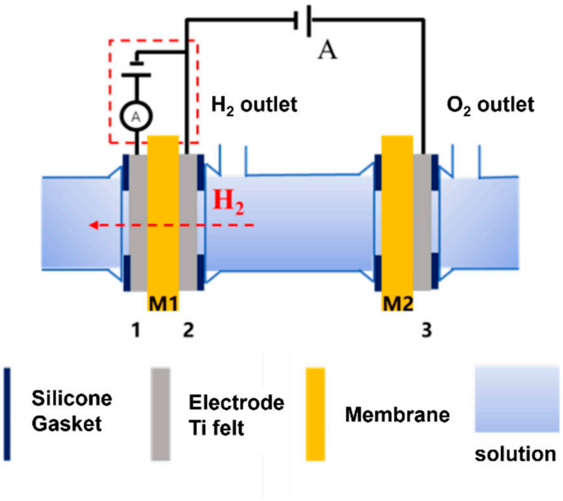

Figure S3. Schematic diagram of the in-situ method for H_2 permeability of the membrane(M1) [1].

The hydrogen permeability of the membranes was determined via an in-situ electrochemical method as described in reference [1]. Figure S3 is a schematic diagram of the measuring device. The negative pole of DC power supply A is connected to electrode 2 (hydrogen-extraction reaction (HER) electrode) to produce H_2. The positive pole of DC power supply A is connected to the electrode 3 oxygen-extraction reaction (OER) electrode to produce O_2. The membrane to be measured, M1, is tightly clamped between electrode 1 (Ti felt, the HOR detection electrode) and electrode 2. The H_2 generated at electrode 2 permeates through the membrane M1 and diffuses into electrode 1. Applying a voltage between electrodes 1 and 2 (electrode 1 is the anode), H_2 undergoes a HOR reaction at electrode 1, generating a HOR current. At the beginning of the reaction, the reaction at both electrodes is controlled by the reaction kinetics, and the HOR current increases with the increase of voltage; ideally, when the diffusion of H_2 controls the HOR, the current value does not increase anymore with the increase of voltage but stays constant. The value of the limiting current of the HOR corresponds to the permeability of H_2 under the measurement conditions. The limiting current within the red dashed box was measured using an electrochemical workstation.

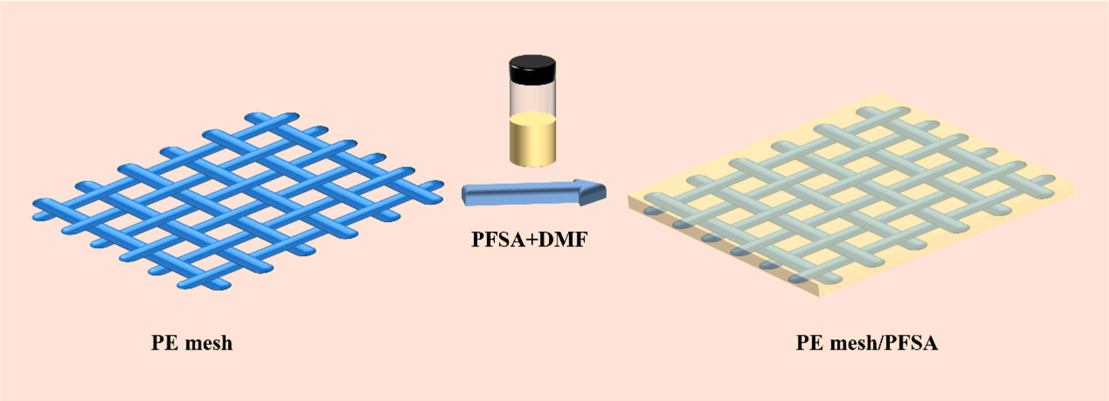

Figure S4. Schematic diagram of PE mesh/PFSA composite membrane preparation process.

1. Li, S.; Song, C.; Xu, L.; Wang, Y.; Zhang, W. In-Situ Measurement of Gas Permeability for Membranes in Water Electrolysis. *Membranes* **2025**, *15*, 147, https://doi.org/10.3390/membranes15050147.
